# The 2039 A/G FSH receptor gene polymorphism influences glucose metabolism in healthy men

**DOI:** 10.1007/s12020-020-02420-3

**Published:** 2020-07-17

**Authors:** Rossella Cannarella, Nicolò Musso, Rosita A. Condorelli, Marco Musmeci, Stefania Stefani, Antonio Aversa, Sandro La Vignera, Aldo E. Calogero

**Affiliations:** 1 Department of Clinical and Experimental Medicine, University of Catania, Catania, Italy; 2 Bio-nanotech Research and Innovation Tower (BRIT), University of Catania, Catania, Italy; 3 Department of Experimental and Clinical Medicine, “Magna Graecia” University, Catanzaro, Italy

**Keywords:** FSH, 2039 A/G FSHR, FSHR polymorphism, Glucose, Metabolism, Insulin

## Abstract

**Objective:**

To assess the role of c. 2039 A/G (p. Asp680Ser) (rs6166) and c. −29 G/A (rs1394205) *follicle-stimulating hormone receptor* (*FSHR*) gene single nucleotide polymorphisms (SNPs) in a cohort of healthy men.

**Methods:**

One-hundred twenty-seven healthy men underwent evaluation of the anthropometric parameters, assessment of metabolic and lipid profile, measurement FSH serum levels, and genotyping of both the aforementioned FSHR SNPs. Data grouped according to the FSHR rs6166 or rs1394205 genotypes underwent to statistical analysis.

**Main results:**

The three groups of men for each FSHR SNP did not differ statistically significantly for body mass index and serum FSH levels. As for *FSHR* rs6166 SNP, glucose levels were significantly lower in men with the GG genotype compared with those with the AA genotype. Men with AG had lower insulin levels and HOMA index values compared with those carrying the genotype AA (*p* < 0.05). The GG group showed a negative correlation between serum FSH levels and insulin and between serum FSH levels and HOMA index (*p* < 0.05). In contrast, men grouped according to the *FSHR* rs1394205 genotype showed no significant difference in blood glucose, serum insulin levels, and HOMA index. The AG group showed a negative correlation between FSH insulin and between serum FSH levels and HOMA index (*p* < 0.05).

**Conclusions:**

Men with the genotype GG of the *FSHR* rs6166 SNP have lower blood glucose levels than those with the AA genotype. Their FSH levels inversely correlated with insulin and HOMA index. In contrast, the genotype *FSHR* rs6166 A/G did not reveal any role of FSH on glucose metabolism in healthy men. The inverse relationship between FSH and insulin or HOMA index in the group with the genotype GG of the *FSHR* rs6166 SNP suggests a possible cross-talk between FSH and insulin.

## Introduction

The follicle-stimulating hormone (FSH) is a glycoprotein made of an α and a β subunit. It exerts its peripheral effects by binding to its receptor (FSHR), a 678 amino acid protein belonging to the G-coupled receptor family, expressed in granulosa and Sertoli cells, where FSH stimulates follicle genesis and spermatogenesis, respectively [1].

The *follicle-stimulating hormone receptor* (*FSHR*) gene, mapping in the 2p16.3 chromosome, contains ten exons and nine introns. The first nine exons encode for the majority of the extracellular domain. The exon 10 encodes for the C-terminal region of the extracellular domain, the transmembrane and the intracellular domains [1]. The HapMap database (http://hapmap.ncbi.nlm.nih.gov) lists more than 900 *FSHR* single nucleotide polymorphisms (SNPs). A large body of evidence has been released on the c. 2039 A/G (p. Asp680Ser) (rs6166) and the c. −29 G/A (rs1394205) *FSHR* SNPs so far. The first maps in the exon 10, which encodes for the transmembrane domain of the FSHR, and it is known to influence the efficiency of signal transduction. In detail, the c. 2039 A/G genotype impacts on the expression of the amino acid 680 and the FSHR Ser680Ser (GG) results to be more resistant to the FSH signal compared with the Asp680Asp (AA) [2]. The c. −29 G/A SNP maps in the *FSHR* gene promoter and influences *FSHR* expression. Particularly, the A allele is associated with a lower (by 56%) transcription activity of the promoter [3].

Classically, the effects of FSH have been restricted to gonads and a large body of evidence of *FSHR* SNPs has been published on fertility in both genders [4–6]. However, recent data point to the existence of extra-gonadal effects of FSH. Accordingly, the *FSHR* is expressed in human bone cells and in human bone marrow adipocytes [7, 8]. Furthermore, FSH has been already addressed as possibly implicated in the postmenopausal changes of bone mineral density [7, 9] and in lipid distribution, adiposity, and metabolism in male patients with hypergonadotropic hypogonadism [8].

To further explore the role that FSH may play in glucose and lipid metabolism in the male gender, we investigated the possible influence of the c. 2039 A/G (p. Asp680Ser) (rs6166) and the c. −29 G/A (rs1394205) *FSHR* SNPs on blood glucose, insulin, total, HDL and LDL cholesterol, triglycerides, on the homeostasis model assessment (HOMA) index and the body mass index (BMI) in a cohort of healthy men.

## Subjects and methods

### Patient selection

The study has been carried out in male patients referring to the Division of Andrology and Endocrinology, University of Catania, for an endocrinologic or andrologic counseling. A detailed medical history was collected and weight and height were assessed for each man. The BMI was calculated using the formula: weight (kg)/[height (m) × height (m)]. A total of 146 men were evaluated for inclusion in this study. The following exclusion criteria were used and this resulted in the exclusion of a number of men that is indicated in parentheses: azoospermia (*n* = 3), tumor (*n* = 2), assumption of metformin, insulin, hypoglycemic or hypocholesterolemic drugs (*n* = 5), diabetes (*n* = 2), use of gonadotoxic drugs (*n* = 2), hypogonadism (*n* = 3), testosterone replacement therapy (*n* = 1), FSH therapy (*n* = 1), major adverse cardiovascular events (*n* = 0), and major comorbilities (*n* = 0). Taking all this into account, 127 Caucasian men from Eastern Sicily were included in the study.

Hormone evaluation was performed by electro chemiluminescence (Hitachi-Roche equipment, Cobas 6000, Roche Diagnostics, Indianapolis, IN, USA). Reference values were as follows: FSH 0.95–11.95 IU/l, glycemia 74–100 mg/dl, insulin 1.9–23 µIU/ml, total cholesterol 0–200 mg/dl, HDL cholesterol 35–55 mg/dl, LDL cholesterol <160 mg/dl, and triglycerides 35–200 mg/dl. The HOMA index was evaluated using the formula: [glycemia (mg/dl) × insulin (µIU/ml)]/405. The upper normal value of HOMA index was 2.5.

### FSHR analysis

Genomic DNA was extracted from blood cells using the PureLink® Genomic DNA Kits (invitrogen Catalog Numbers K1821–04) for purification of genomic DNA according to the manufacturer’s instructions. The concentration and the quality of the DNA was determined using a ND-1000 spectrophotometer (NanoDrop, Thermo Scientific, USA). Allelic Discrimination was performed with TaqMan assay in order to show a different genotyping distribution of *FSHR* polymorphism. Probes and primers for rs6166 *FSHR* and rs1394205 *FSHR* polymorphisms were chosen on https://www.thermofisher.com/it/en/home/life-science/pcr/real-time-pcr/real-time-pcr-assays/snp-genotyping-taqman-assays.html?SID=fr-taqman-2. The reaction was carried out according to manufacturer’s instructions (cod 4371355, Applied Biosystems, CA, USA). Each DNA sample was analyzed in triplicate [10]. Allelic Discrimination real-time PCR analysis was performed using LightCycler® 480 System (Roche Molecular Systems, Inc).

### Statistical analysis

The normality of the variables was evaluated with the Shapiro–Wilk test. Descriptive statistical results have been reported as mean ± standard deviation (SD) for not-skewed variables. Statistical analysis was performed by one-way analysis of variance, followed by the Duncan’s Multiple Range Test, using SPSS 22.0 for Windows (SPSS Inc., Chicago, IL, USA). For correlation analysis, the Pearson or the Kendall tests were used, according to the data distribution. A *p* value < 0.05 was accepted as statistically significant.

## Results

The number and the prevalence (in parentheses) of *FSHR* rs6166 genotypes were: 33 (26.0%), 63 (49.6%), and 31 (24.4%) for AA, AG, and GG, respectively. For the genotype *FSHR* rs1394205, they were: 14 (11.0%), 38 (29.9%), and 75 (59.1%) for AA, AG, and GG genotypes, respectively. The *FSHR* allelic frequencies and the estimated *FSHR* haplotype frequencies found in this cohort were consistent with previously published data from unrelated Caucasian populations [9, 11–13].

Data were normally distributed. Anthropometric data and biochemical parameters of men grouped according to the *FSHR* rs6166 or rs1394205 genotype are shown in Table 1. No significant difference in all parameters was observed between men classified according to the different genotype (*p* > 0.05 in all cases), with the exception of total cholesterol, which was higher in men with *FSHR* rs1394205 AA genotype compared with those with the AG genotype (*p* < 0.05). According to their BMI, 42.2% of patients were overweight, 20% obese; 8.3% had hyperglicemia, 20.5% insulin resistance, and 26.3% hypercholesterolemia. The prevalence of these features did not differ after classification by the *FSHR* genotype.

**Table 1:** Antropometric data and biochemical parameters in 127 healthy men classified according to rs6166 or rs1394205 polymorphisms of the FSH receptor gene

Parameters	rs6166			rs1394205		
	AA	AG	GG	AA	AG	GG
Number	33	63	31	14	38	75
Age (years)	37.0 ± 8.7	36.6 ± 9.2	35.7 ± 7.6	40.4 ± 8.5	36.9 ± 10.9	35.4 ± 7.4
BMI (kg/m^2^)	27.0 ± 4.9	27.3 ± 5.4	26.5 ± 3.8	27.0 ± 3.4	26.6 ± 5.5	27.1 ± 4.9
Glycaemia (mg/dl)	90.5 ± 7.4	86.2 ± 8.6	82.2 ± 5.5*	87.4 ± 12.7	88.2 ± 7.9	85.2 ± 7.8
Insulin (µIU/ml)	11.0 ± 4.8	7.3 ± 3.0*	9.0 ± 1.4	7.5 ± 2.9	8.1 ± 3.7	9.4 ± 3.5
HOMA index	2.5 ± 1.3	1.6 ± 0.7*	1.8 ± 1.0	1.6 ± 0.9	1.8 ± 0.9	2.0 ± 1.0
Cholesterol (mg/dl)	184.7 ± 46.6	179.4 ± 36.6	178.6 ± 30.1	208.5 ± 45.5	174.3 ± 28.6*	184.1 ± 37.7
HDL cholesterol (mg/dl)	46.8 ± 14.2	50.4 ± 13.6	47.0 ± 9.3	52.2 ± 12.8	49.7 ± 10.0	46.9 ± 11.7
LDL cholesterol (mg/dl)	134.4 ± 46.6	115.8 ± 32.1	120.9 ± 31.1	145.3 ± 40.7	115.7 ± 39.8	121.6 ± 32.7
Triglycerides (mg/dl)	88.6 ± 49.3	81.4 ± 64.4	93.1 ± 40.2	165.8 ± 33.7	92.5 ± 57.2	92.2 ± 63.1
FSH (IU/l)	5.0 ± 3.2	4.5 ± 2.9	3.9 ± 2.5	4.0 ± 2.3	4.5 ± 3.0	4.4 ± 2.8

Data are expressed as mean ± standard deviation for continual variables. The *p* values were calculated using ANOVA for continuous variables

*FSH* follicle-stimulating hormone, *BMI* body mass index, *HOMA* homeostasis model assessment

*p < 0.05 vs. AA (ANOVA followed by Duncan Multiple Range Test)

**Fig. 1:**
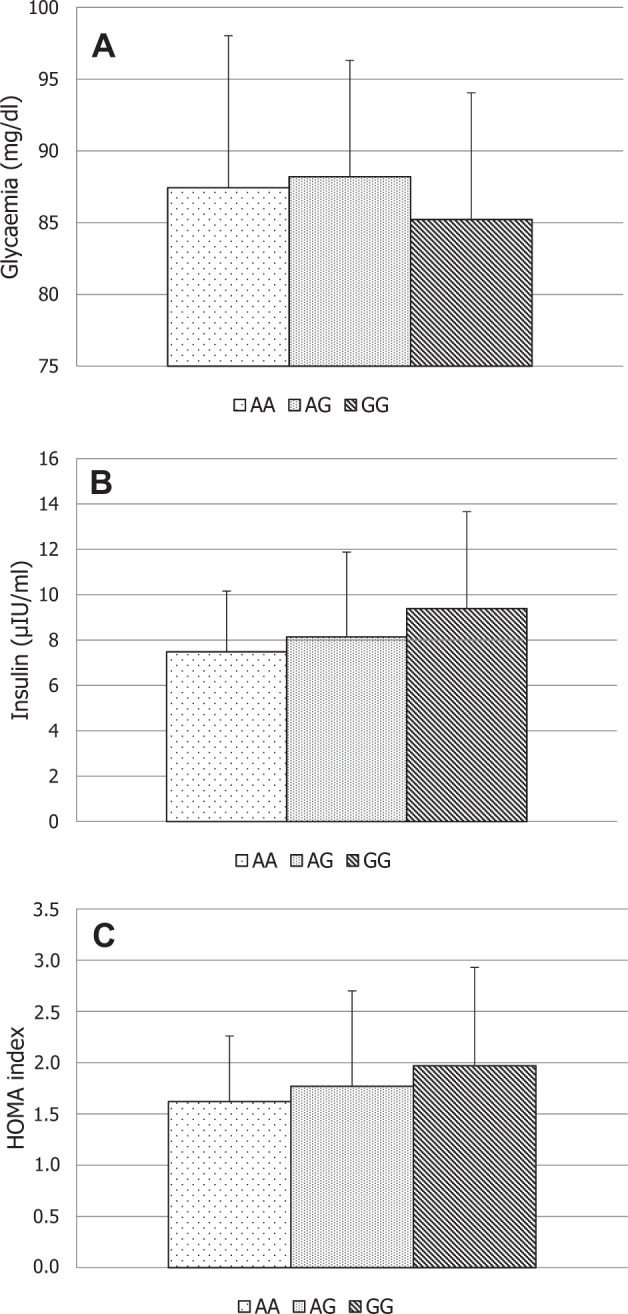
Glycaemia, insulin, and HOMA index classified according to the rs1394205 *FSHR* genotype. No significant difference was found in AA, AG, and GG groups for glycaemia (**a**), insulin (**b**), and HOMA index (**c**)

Serum glucose levels were lower in men with *FSHR* rs6166 GG vs. men with the AA genotype. Men with AG SNP had lower insulin levels and HOMA index values (Table 1) compared with AA (*p* < 0.05). Men grouped for the *FSHR* rs1394205 genotype did not show any difference in glycaemia, insulin, and HOMA index (Fig. 1).

No significant correlation was found between FSH and glycaemia (*r* = 0.01), insulin (*r* = −0.26) or HOMA index (*r* = −0.19) in the entire cohort. Correlation analysis in men grouped by *FSHR* rs6166 or rs1394205 genotypes are shown in Table 2. A significant negative correlation between FSH and insulin and FSH and HOMA index was observed in men with the genotype *FSHR* rs6166 GG and for the genotype *FSHR* rs1394205 AG. All the other correlations were not significant.

**Table 2:** Correlation analysis of glycaemia, insulin, and HOMA index with the rs6166 or rs1394205 polymorphisms of the FSH receptor gene, in 127 healthy men

Parameters	rs6166			rs1394205		
	AA	AG	GG	AA	AG	GG
FSH vs. glycaemia	*r* = −0.29	*r* = 0.07	*r* = 0.12	*r* = 0.11	*r* = −0.20	*r* = 0.08
FSH vs. insulin	*r* = −0.02	*r* = −0.16	*r* = −0.69^*^	*r* = −0.31	*r* = −0.67^*^	*r* = −0.19
FSH vs. HOMA index	*r* = −0.11	*r* = −0.07	*r* = −0.71^*^	*r* = −0.17	*r* = −0.70^*^	*r* = −0.07

*HOMA* homeostasis model assessment, *FSH* follicle-stimulating hormone

*p ≤ 0.05

## Discussion

The results of the present study showed that the polymorphism rs6166 in exon 10 of the *FSHR* gene influences significantly blood glucose levels in healthy men. Particularly, the GG genotype, encoding for a less efficiently transducing FSHR protein, is associated with lower glucose levels compared to the AA one, a SNP that more efficiently transduce the FSH signal. Since the groups resulted to have a similar age, BMI and FSH levels, these findings suggest that the *FSHR* gene plays a role on glucose metabolism in men. The significant lower insulin levels and HOMA index in men with the AG genotype compared those with AA suggest a sort of compensatory mechanism to avoid hyperglycemia, which is not observed in men with GG due to the normal glucose levels. Interestingly, an inverse relationship between FSH and insulin levels or HOMA index was found in men with the genotype GG. Furthermore, no effect on such parameters was observed when men were classified according to the rs1394205 genotype, aside a significant indirect correlation between FSH and insulin and a negative trend between FSH and HOMA index in the AG group.

Scanty evidence has been published on the impact of FSH on glucose metabolism so far. A preclinical study reported that female mice FSHR gene deficient (*Fshr*^+/−^ mice) do not develop obesity as wild-type ovariectomized mice do. These effects were also reported in male *Fshr*^+/−^ mice. Furthermore, the administration of a blocking antibody against FSH was found to efficiently induce thermogenic adipose tissue and to reduce body fat in both sexes [14]. This indicates that FSH may be involved in the development of obesity. In addition, the FSHR has been found expressed in pancreatic ß cells in animals but its function in this district is still unclear [15].

A recent clinical study investigated whether FSH may impact on glucose and lipid metabolism in a large number of patients (more then 300) with hypergonadotropic hypogonadism, including patients with Klinefelter syndrome. Remarkably, the authors reported that FSH could enhance soluble-RANKL (sRANKL) secretion from human adipocytes. sRANKL levels, in turn, are negatively associated with fat percentage, fasting insulin, and glucose [8]. sRANKL has been shown to decrease pancreatic ß cell proliferation and to decrease glucose tolerance [16]. Accordingly, the administration of the sRANKL inhibitor denosumb improves glucose tolerance, but not HbA1c levels in nondiabetic women [17, 18]. Thus, the association between FSH and glucose metabolism may be indirect being mediated by the FSH-induced sRANKL production.

The inverse relationship between FSH and insulin or HOMA index observed in men with the GG genotype of the *FSHR* rs6166 SNP suggests the hypothesis that FSH and insulin may share a common molecular signaling. Generally, tyrosine kinase receptors (e.g., insulin receptor or insulin-like growth factor receptor) are able to influence the FSHR signaling [19–21]. In this regard, the insulin receptor substrate 1, which represents a co-factors linked to tyrosine kinase receptors, seems to play a role in the pathogenesis of insulin resistance [22, 23]. Interestingly, it has been described as the hub that links FSH signaling pathway and the tyrosine kinase receptor-dependent cascade [24, 25]; thus relating FSHR to the insulin receptor. The possible cross-talk between FSH and insulin has been further investigated in an in vitro study on porcine prepubertal Sertoli cells aimed to evaluate the secretion of anti-Müllerian hormone (AMH) and inhibin in response to FSH and insulin. The results showed that insulin significantly decreased FSH-stimulated AMH and inhibin secretion, suggesting that insulin may likely interfere with the FSH signaling [26]. In line with this evidence, a recent clinical study reported that insulin is able to decrease the response of granulosa cells to FSH stimulation in women with obesity-related infertility during in vitro fertilization [27]. Unfortunately, clinical evidence is very limited in men, due to the small duration of FSH stimulation of the poorly standardized protocols used for the treatment of infertile male patients [28]. FSH administration to patients with oligozoospermia, normal serum FSH levels, and insulin resistance showed to increase its efficacy on sperm parameters and fertility outcome when it was co-administered with metformin [29]. These findings add further evidence on the possible interference of insulin in FSH signaling also in men. Conversely, FSH may somehow influence insulin pathway and glucose metabolism, which may be dimly supported by the significantly increase of FSH serum levels in diabetic patients, as results of meta-analysis have shown [30]. In addition, an inverse correlation between FSH and insulin, and FSH and HOMA index were found in the rs1394205 AG group. The reason of this result is hardly to be interpreted. This strengthen the importance of fully addressing the molecular relationship between FSHR and insulin.

Finally, the results of the present study expand the view of the possible clinical applications of *FSHR* SNPs assessment. A possible *FSHR* SNPs usefulness has been classically searched on fertility so far. However, the research promoted after the identification of FSH effects on bone tissue has led to studies aimed at exploring its protective role against the development of osteoporosis in women with the *FSHR* rs6166 GG genotype; thus supporting extra-gonadal effects that FSH may likely have [9]. Similarly, the importance of *FSHR* SNPs in the metabolic field needs to be further addressed.

Our results need to be taken with care due to the relatively small sample size examined. Hence, further studies on greater cohorts are warranted.

In conclusion, we found significantly lower blood glucose levels in men with the GG genotype of the FSHR rs6166 SNP, and significantly lower insulin levels and HOMA index in AG compared with AA men. Furthermore, FSH serum levels correlated inversely with insulin levels and the HOMA index in men with GG, no effects were observed on these parameters when men were classified according to the rs1394205 genotype, although a significant indirect correlation between FSH and insulin and a negative trend between FSH and HOMA index was found in the AG group. *FSHR* rs6166 A/G genotypization could reveal the impact of FSH on glucose metabolism in healthy men. The inverse relationship of FSH with insulin and the HOMA index in the *FSHR* rs6166 GG group may support the existence of a cross-talk between FSH and insulin. Accordingly, the higher is insulin, the lower is FSH, thus reflecting the importance of treating insulin resistance prior to FSH administration in oligozoospermic patients [29]. Molecular mechanisms underlying the interaction between FSH and insulin are still matter of debate. Unpublished data support that incubation with insulin down-regulates FSH receptor in an in vitro culture of porcine Sertoli cells. The in vivo findings of a recently published study [29] may somehow reflect this molecular mechanism. With this in mind, the inverse relationship between FSH and insulin and between FSH and HOMA index in the GG group (encoding for a more resistant *FSHR* to signal transduction) could confirm the occurrence of a molecular interaction between insulin and the FSHR. Clearly, this topic represents a black hole and deserves further investigation.
